# Adolescent alcohol use and parental and adolescent socioeconomic position in six European cities

**DOI:** 10.1186/s12889-017-4635-7

**Published:** 2017-08-08

**Authors:** Marina Bosque-Prous, Mirte A. G. Kuipers, Albert Espelt, Matthias Richter, Arja Rimpelä, Julian Perelman, Bruno Federico, M. Teresa Brugal, Vincent Lorant, Anton E. Kunst

**Affiliations:** 1 Agencia de Salut Pública de Barcelona, Plaça Lesseps 1, 08023 Barcelona, Spain; 2 Institut d’Investigació Biomèdica Sant Pau (IIB Sant Pau), Carrer Sant Antoni Maria Claret 167, 08025 Barcelona, Spain; 3 Universitat Pompeu Fabra, Carrer Doctor Aiguader 80, 08003 Barcelona, Spain; 4 Department of Public Health, Academic Medical Center, University of Amsterdam, 1100 DD Amsterdam, Amsterdam, the Netherlands; 5 Centros de Investigación Biomédica en Red. Epidemiología y Salud Pública (CIBERESP), Calle Melchor Fernández Almagro 3-5, 28029 Madrid, Spain; 6 Departament de Psicobiologia i Metodologia en Ciències de la Salut, Facultat de Psicologia, Universitat Autònoma de Barcelona, Campus UAB Bellaterra, 08193 Cerdanyola del Vallès, Spain; 7 Institute of Medical Sociology (IMS), Medical Faculty, Martin Luther University Halle-Wittenberg, Magdeburger Str. 8, 06112 Halle (Saale), Germany; 8 School of Health Sciences and PERLA - Tampere Centre for Childhood, Youth and Family Research, University of Tampere, 33014 Tampere, Finland; 9 Department of Adolescent Psychiatry, Tampere University Hospital, -33521 Tampere, FI Finland; 10 Escola Nacional de Saúde Pública, Universidade NOVA de Lisboa, 1600-560 Lisbon, Portugal; 11 Centro de Investigação em Saúde Pública, 1600-560 Lisbon, Portugal; 12 Department of Human Sciences, Society and Health, University of Cassino and Southern Lazio, 03043 Cassino, Italy; 13 Institute of Health and Society, Université Catholique de Louvain, 3016-1200 Brussels, Belgium

**Keywords:** Alcohol drinking, Adolescence, Socioeconomic factors, Europe

## Abstract

**Background:**

Many risk behaviours in adolescence are socially patterned. However, it is unclear to what extent socioeconomic position (SEP) influences adolescent drinking in various parts of Europe. We examined how alcohol consumption is associated with parental SEP and adolescents’ own SEP among students aged 14–17 years.

**Methods:**

Cross-sectional data were collected in the 2013 SILNE study. Participants were 8705 students aged 14–17 years from 6 European cities. The dependent variable was weekly binge drinking. Main independent variables were parental SEP (parental education level and family affluence) and adolescents’ own SEP (student weekly income and academic achievement). Multilevel Poisson regression models with robust variance and random intercept were fitted to estimate the association between adolescent drinking and SEP.

**Results:**

Prevalence of weekly binge drinking was 4.2% (95%CI = 3.8–4.6). Weekly binge drinking was not associated with parental education or family affluence. However, weekly binge drinking was less prevalent in adolescents with high academic achievement than those with low achievement (PR = 0.34; 95%CI = 0.14–0.87), and more prevalent in adolescents with >€50 weekly income compared to those with ≤€5/week (PR = 3.14; 95%CI = 2.23–4.42). These associations were found to vary according to country, but not according to gender or age group.

**Conclusions:**

Across the six European cities, adolescent drinking was associated with adolescents’ own SEP, but not with parental SEP. Socio-economic inequalities in adolescent drinking seem to stem from adolescents’ own situation rather than that of their family.

**Electronic supplementary material:**

The online version of this article (doi:10.1186/s12889-017-4635-7) contains supplementary material, which is available to authorized users.

## Background

Alcohol consumption ranks among the top five risk factors for disease, disability and death throughout the world [1]. Adolescence is a period in which many risk behaviours including alcohol consumption are initiated [2]. Prevalence rates among European adolescents vary from 6 to 23% for weekly alcohol consumption [3, 4], and from 27% to 70% for monthly binge drinking (i.e. drinking large amounts of alcohol in a single occasion) [5]. In adolescents, frequent alcohol use and binge drinking often co-occur with other problem behaviours (e.g. academic problems, use of other substances, delinquent behaviour, driving or riding under the influence of alcohol), which may pose significant challenges to making a successful transition from adolescence to adulthood [6–8]. Moreover, alcohol use in adolescence predicts alcohol use and alcoholism in adulthood [9].

Many risk behaviours in adolescence are socially patterned. Several studies have assessed the possible association between parental socioeconomic position (SEP) and adolescent alcohol consumption. These studies yielded inconsistent results [10–12]. According to a review of the association between SEP and adolescent alcohol consumption, most of the high quality studies found no significant association, two studies reported negative associations and four studies found positive associations [10]. Differences in results could be due to the use of different SEP indicators (e.g. parental education, parental occupation or family income), which may measure different and complementary dimensions of SEP [12]. Inconsistencies between studies might also be explained by variations in national contexts, including differences in patterns and meanings of use of alcohol [10, 11]. Some studies have also suggested that the relationship between parental SEP and adolescent drinking varies with age and gender [13, 14].

The lack of a consistent association between the parental SEP and adolescent alcohol use could be due to adolescence being a period of decreasing parental influence on risk behaviours [15]. Thus, measures more closely related to the adolescent such as their academic performance or their financial resources might play a more important role in adolescent drinking behaviour than socioeconomic characteristics of the parents. Previous studies showed that higher adolescent income was associated with regular alcohol use and consumption of larger amounts [16, 17], and academic achievement was inversely associated with alcohol consumption [18].

The aim of this study was to estimate the association between adolescent drinking and parental SEP and adolescents’ own SEP among students aged 14–17 years, and to analyse which SEP indicator has a stronger association with adolescent drinking. We additionally explored whether the associations varied according to country, gender and age group. The particular value of this study lies in analysing multiple SEP indicators at parental and adolescent level at the same time, and in using a large dataset covering cities from six different European countries.

## Methods

### Design and study population

The study used a cross-sectional design with data from the SILNE (Smoking Inequalities: Learning from Natural Experiments) survey, conducted in 2013. The SILNE survey was primarily aimed at studying socioeconomic inequalities in smoking in different European cities [19], but it also included variables on alcohol consumption. The SILNE survey sample included 50 secondary schools from six European cities: Namur (Belgium), Tampere (Finland), Hannover (Germany), Latina (Italy), Amersfoort (the Netherlands) and Coimbra (Portugal) (*n* = 11,015; 79.4% response rate). All data were self-reported by the students. Ethical approval was obtained in all countries. Methodological details on the SILNE survey have been published elsewhere [19]. For this study, we excluded students under 14 and over 17 (*n* = 424) and those with missing information on both alcohol measures (*n* = 12), demographic variables (*n* = 178) and parental and adolescent SEP variables (*n* = 1696). The final sample size was of 8705 students.

### Measures

#### Dependent variables

The dependent variable was weekly binge drinking, defined as drinking 5 or more alcoholic drinks on one occasion at least once a week in the previous 12 months (yes/no). Moreover, to describe adolescent alcohol consumption, weekly alcohol consumption (i.e. drinking at least one alcoholic beverage per week in the prior 12 months) was also used to perform a sensitivity analysis. Information on weekly binge drinking and weekly alcohol consumption was available for 8541 and 8652 individuals, respectively.

#### Independent variables

##### Parental SEP

We measured two different indicators of parental SEP: parental education level and family affluence, which have been previously used [4, 10, 12, 20]. Parental education level was measured using a country-specific classification that was collapsed into three categories: low (primary school or lower level of secondary school), middle (completed secondary school or lower level college) or high educational level (university degree). For each student, the education level of the parent with the highest attainment was used. Family affluence was measured with the Family Affluence Scale (FAS) [21], which was constructed with 4 questions of the SILNE questionnaire: 1) Does your family own a car, van or truck?; 2) Do you have your own bedroom?; 3) How many computers/laptops/tablets does your family own?; 4) During the past 12 months, how many times did you travel away on holiday with your family? Question 2 was rated from 0 to 1 and questions 1, 3 and 4 were rated from 0 to 2, resulting in an index with values from 0 to 7. High scores represented higher family affluence. FAS was categorised due to low numbers at the extremes of the scale. For reasons of statistical power, we used different cut-offs for the total sample analysis than for stratified analyses.

##### Adolescent SEP

We measured two different indicators of adolescents SEP, the amount of money students had available to spend on themselves each week and the academic achievement, which have been used in previous studies [22, 23]. Student weekly income (i.e. the amount of money the adolescent usually had each week to spend on themselves or to save from pocket money and jobs) was divided into five categories: €0–5, €6–10, €11–20, €21–50 or >€50. Due to low numbers in some categories in the stratified analysis, we used a variable with only three groups: €0–5, €6–20 and >€20. Adolescent academic achievement was based on the student’s marks during the previous school year, (classified in five categories: insufficient, low, average, good or high achievement. For the analysis stratifying by country, we used a variable with only three categories: low (insufficient and low marks), average and good (good and high marks).

##### Other independent variables

We measured confounders likely to predict adolescent drinking, such as age (in years), gender (male vs. female) and migrant background [24]. We distinguished between native background, mixed background (one parent born in another country than the country of residence), and migrant background (both parents born in a foreign country). In the stratified analysis age was classified into two groups: younger students (14–15 years old) and older students (16–17 years old).

### Statistical analysis

First, we described the distribution of the independent variables in the sample and estimated the age-adjusted prevalence of weekly binge drinking and weekly alcohol consumption for all subgroups. We also tested for polychoric correlations between the dependent variables, as the variables were categorical [25]. As we found that weekly binge drinking and alcohol consumption were highly correlated (*r* = 0.88), we decided to focus the study on weekly binge drinking and conduct a sensitivity analysis with weekly alcohol consumption as the dependent variable.

We calculated polychoric correlations between the different adolescent and parental SEP variables to assess potential multicollinearity in the regression model. We found that the correlations between these variables were weak (between −0.10 and +0.21). To estimate the associations between weekly binge drinking and parental education, family affluence, student weekly income and academic achievement, we fit multilevel Poisson regression models with robust variance and random intercept (2 levels: student and school). These models yielded Prevalence Ratios (PR) and their 95% confidence intervals (95%CI). We tried to fit a three-level model including the city as the highest level, but the model fit poorly due to the fact that school variability explained the differences between countries and also because there were few countries in the study. We used Poisson regression models with robust variance instead of logistic regression models as they yielded PR, which have the advantage over Odds Ratios to have a concrete interpretation even when prevalence rates are high [26].

We fit several models in various steps. In each step, we adjusted for gender, age and migrant background. In step 1 we estimated the variance among schools. In step 2 we fit several models including in each model only one SEP measure (parental education, family affluence, student weekly income or academic achievement). In step 3 we included all SEP measures in one model simultaneously. We conducted a sensitivity analysis using multiple imputation in addition to the complete-case analysis approach in order to assess whether the method used to deal with missing data influenced the results.

To test whether the associations were consistent among gender, age and country, we also fit the same regression models per gender, age and country. All analyses were conducted using STATA 13.

## Results

Table 1 presents the distribution of the study population and the prevalence of weekly binge drinking and weekly alcohol consumption for each of the independent variables. The mean age of participants was 15.2 years and 53.5% were girls. Overall, age-adjusted prevalence of weekly binge drinking and alcohol consumption were 4.2% (95%CI = 3.8–4.6) and 11.3% (95%CI = 10.7–12.0), respectively, with relevant variations between countries. The prevalence of weekly binge drinking varied from 1.1% (95%CI = 0.7–1.9) in Finland to 6.1% (95%CI = 5.1–7.3) in Belgium, and the prevalence of weekly alcohol consumption varied from 2.1% (95%CI = 1.4–3.1) in Finland to 19.8% (95%CI = 18.1–21.7) in Italy. We did not observe consistent differences in the prevalence of alcohol consumption between categories of parental SEP indicators, whereas prevalence rates were clearly higher in adolescents with lower academic achievement and higher weekly income. The age-adjusted prevalence of weekly binge drinking in adolescents who received €0–5/week and >€50/week was 2.4% and 10.7%, respectively. In adolescents whose academic achievement was the lowest and the highest, the age-adjusted prevalence of weekly binge drinking was 7.0% and 1.5%, respectively.

**Table 1:** Distribution of the individual independent variables and age-adjusted prevalence of weekly binge drinking and weekly alcohol consumption (i.e. drinking at least one alcoholic beverage per week) among 14–17 years-old students from 6 European cities participating in the SILNE survey, 2013

	All participants		Weekly binge drinking		Weekly alcohol consumption	
	N	%	Age-adjusted prevalence	95%CI	Age-adjusted prevalence	95%CI
Gender						
Female	4658	53.5	2.9	(2.4–3.5)	8.4	(7.7–9.3)
Male	4047	46.5	5.6	(4.9–6.3)	14.6	(13.5–15.7)
City (country)						
Namur (Belgium)	1653	19.0	6.1	(5.1–7.3)	15.7	(14.1–17.4)
Tampere (Finland)	1016	11.7	1.1	(0.7–1.9)	2.1	(1.4–3.1)
Hannover (Germany)	1060	12.2	4.9	(3.6–6.7)	7.6	(6.0–9.6)
Latina (Italy)	1874	21.5	5.7	(4.7–6.8)	19.8	(18.1–21.7)
Amersfoort (Netherlands)	1548	17.8	4.6	(3.5–5.9)	13.8	(12.0–15.7)
Coimbra (Portugal)	1554	17.8	1.9	(1.4–2.6)	4.7	(3.9–5.8)
Migrant background						
Native	6988	80.3	4.3	(3.8–4.8)	12.1	(11.4–12.9)
Mixed couples	913	10.5	4.8	(3.6–6.5)	10.8	(8.9–12.9)
Both parents immigrants	804	9.2	3.1	(2.1–4.5)	6.4	(4.9–8.2)
Parental education level						
Low level	1177	13.5	5.3	(4.1–6.8)	11.5	(9.7–13.6)
Middle level	3497	40.2	4.7	(4.0–5.5)	12.7	(11.5–13.9)
High level	4031	46.3	3.7	(3.1–4.4)	10.8	(9.8–11.9)
Family Affluence Scale						
0–2	837	9.6	5.5	(4.0–7.4)	11.4	(9.3–13.7)
3	1360	15.6	4.1	(3.1–5.2)	9.7	(8.3–11.4)
4	2253	25.9	3.7	(3.0–4.5)	9.3	(8.2–10.5)
5	1522	17.5	3.8	(2.9–4.9)	12.0	(10.5–13.8)
6–7	2733	31.4	4.5	(3.7–5.4)	13.7	(12.4–15.1)
Academic achievement						
Insufficient (<50%)	313	3.6	7.0	(4.9–10.0)	14.2	(11.0–18.1)
Low (50–59%)	1117	12.8	5.5	(4.3–7.0)	14.0	(12.1–16.2)
Average (60–69%)	3616	41.5	4.9	(4.2–5.6)	12.8	(11.8–14.0)
Good (70–84%)	2784	32.0	3.2	(2.6–3.9)	9.8	(8.7–10.9)
High (>85%)	875	10.1	1.5	(0.9–2.6)	5.3	(4.0–6.9)
Student weekly income						
0–5 €	2063	23.7	2.4	(1.8–3.1)	6.9	(5.9–8.0)
6–10 €	2084	23.9	2.0	(1.5–2.7)	7.3	(6.3–8.4)
11–20 €	2070	23.8	3.8	(3.1–4.7)	11.3	(10.0–12.7)
21–50 €	1623	18.7	5.9	(4.8–7.2)	15.5	(13.8–17.3)
> 50 €	865	9.9	10.7	(8.7–13.1)	22.8	(20.1–25.9)
Total	8705	100.0	8541		8652	
Total prevalence			4.2	(3.8–4.6)	11.3	(10.7–12.0)

The results of the multilevel Poisson regression confirm the findings of the descriptive analysis (Table 2). When adjusted by the control variables (age, gender, migrant background and school) and for other SEP variables, we did not find an association between parental SEP indicators and adolescent alcohol consumption, but we found a dose-response type association between weekly binge drinking and adolescent SEP. Adolescents with high academic achievement were less likely to binge drink weekly than those with insufficient academic achievement (PR = 0.34; 95%CI = 0.14–0.87). The association found for student weekly income was in the opposite direction: adolescents with a high weekly income (>€50/week) had a higher risk of weekly binge drinking than those with a low weekly income (≤€5/week) (PR = 3.14; 95%CI = 2.23–4.42). It is important to highlight that the PR for adolescent SEP did not change in the fully adjusted model, which means that parental SEP did not act as a confounder in the relationship between adolescent SEP and alcohol use.

**Table 2:** Prevalence ratios (PR) of binge drinking estimated with multilevel Poisson regression models with robust variance among 14–17 years-old students from 6 European cities participating in the SILNE survey, 2013

	Step 2		Step 3	
	PR	95%CI	PR	95%CI
Parental education level				
Low level	1		1	
Middle level	0.96	(0.75–1.23)	0.93	(0.73–1.19)
High level	0.95	(0.64–1.42)	0.93	(0.64–1.35)
Family Affluence Scale (FAS)				
0–2	1		1	
3	0.93	(0.60–1.44)	0.93	(0.59–1.46)
4	0.95	(0.63–1.41)	0.89	(0.59–1.34)
5	0.96	(0.64–1.43)	0.89	(0.58–1.39)
6–7	1.09	(0.74–1.61)	0.98	(0.66–1.46)
Academic achievement				
Insufficient (<50%)	1		1	
Low (50–59%)	0.75	(0.42–1.33)	0.73	(0.41–1.31)
Average (60–69%)	0.67	(0.36–1.27)	0.66	(0.36–1.21)
Good (70–84%)	0.50	(0.28–0.88)	0.49	(0.29–0.83)
High (>85%)	0.34	(0.13–0.91)	0.34	(0.14–0.87)
Student weekly income				
0–5 €	1		1	
6–10 €	0.93	(0.64–1.38)	0.95	(0.65–1.38)
11–20 €	1.59	(1.11–2.29)	1.55	(1.07–2.24)
21–50 €	2.14	(1.48–3.11)	2.12	(1.47–3.05)
> 50 €	3.10	(2.23–4.30)	3.14	(2.23–4.42)
Variability (% change in variability)^a^			0.458	(10.3)

Step 2 included weekly binge drinking, one SEP indicator and was adjusted by age, gender and migrant background in level 1 and school in level 2. Step 3 also included all SEP indicators

^a^Variability of the empty model (step 1), which included only weekly binge drinking was 0.511. % change in variability was calculated using the following formula: [(variability step 1 - variability current step)/(variability step 1)]×100

The results stratified by gender and age group are presented in Additional file 1: Table S1 and Additional file 2: Table S2, respectively. We did not find significant differences between groups, although the associations seemed stronger in girls and in younger students.

Table 3 shows the results of the analyses stratified by country. In general, country-specific results were similar to those for the whole sample. However, we found some differences between countries. Parental SEP measures were not associated with adolescent drinking in five countries. Italian students from higher affluent families were at lower risk of weekly binge drinking (PR = 0.69; 95%CI = 0.54–0.88). In addition, academic achievement and student weekly income were associated with binge drinking in 2 and 4 out of 6 cities studied, respectively. In the German city, the association with academic achievement seemed to be in the opposite direction to the other five cities, but it was not significant. The association between student weekly income and binge drinking in the Dutch and the Finish cities was not statistically significant, but the point estimates were in the same direction to the other countries.

**Table 3:** Prevalence of binge drinking and prevalence ratios for the socioeconomic position (SEP) variables by country, estimated with multilevel Poisson regression models with robust variance among 14–17 years-old students from 6 European cities participating in the SILNE survey, 2013

	Namur (Belgium)			Tampere (Finland)			Hannover (Germany)			Latina (Italy)			Amersfoort (Netherlands)			Coimbra (Portugal)		
	%	PR	95%CI	%	PR	95%CI	%	PR	95%CI	%	PR	95%CI	%	PR	95%CI	%	PR	95%CI
Parental education level																		
Low level	6.6	1		no observations			2.8	1		7.4	1		5.9	1		4.1	1	
Middle level	6.5	0.87	(0.71–1.07)	1.8	1		5.4	2.11	(0.40–11.15)	5.1	0.83	(0.61–1.13)	4.2	0.74	(0.32–1.71)	3.4	0.83	(0.52–1.32)
High level	7.7	1.26	(0.71–2.25)	0.6	0.70	(0.21–2.36)	3.1	1.47	(0.33–6.59)	4.3	0.85	(0.53–1.35)	2.9	0.63	(0.26–1.54)	2.0	0.51	(0.17–1.50)
Family Affluence Scale (FAS)																		
0–4	6.3	1		1.3	1		4.6	1		6.3	1		3.5	1		2.7	1	
5–7	8.3	1.18	(0.92–1.52)	1.1	0.95	(0.27–3.37)	3.0	0.75	(0.44–1.25)	4.7	0.69	(0.54–0.88)	3.7	1.20	(0.77–1.86)	3.2	1.31	(0.77–2.22)
Academic achievement																		
Low (<60%)	9.2	1		4.2	1		3.3	1		14.2	1		4.3	1		6.5	1	
Average (60–69%)	7.9	0.92	(0.44–1.91)	0.3	0.06	(0.01–0.54)	4.9	1.62	(0.67–3.94)	7.9	0.72	(0.39–1.30)	4.1	1.02	(0.66–1.56)	2.4	0.47	(0.17–1.26)
Good (>70%)	5.2	0.68	(0.45–1.02)	no observations			2.9	1.55	(0.66–3.66)	2.6	0.30	(0.11–0.78)	2.8	0.93	(0.61–1.40)	1.8	0.49	(0.18–1.35)
Student weekly income																		
0–5 €	4.1	1		no observations			1.3	1		3.3	1		2.3	1		2.1	1	
6–20 €	5.4	1.29	(0.90–1.85)	0.9	1		1.9	1.34	(0.30–6.04)	5.4	1.51	(0.92–2.50)	1.2	0.43	(0.13–1.48)	2.7	1.31	(0.43–3.96)
> 20 €	12.1	2.51	(1.49–4.24)	2.2	1.94	(0.94–4.01)	6.4	3.23	(1.00–10.49)	10.5	2.50	(1.28–4.87)	6.2	1.35	(0.63–2.90)	6.9	2.89	(1.29–6.47)

Each model included weekly binge drinking, all SEP indicators and was adjusted by age, gender and migrant background in level 1 and school in level 2

*%* prevalence; *PR* prevalence ratio; *95%CI* 95% confidence interval

Finally, in a sensitivity analysis, we repeated these analyses using weekly alcohol consumption as the dependent variable. The results of the multilevel Poisson regression models for weekly alcohol consumption are shown in Additional file 3: Table S3. The stratified analyses are shown in Additional file 4: Table S4, Additional file 5: Table S5 and Additional file 6: Table S6. The estimates for both alcohol measures were similar in all analyses. When conducting a sensitivity analysis with multiple imputation of missing values, we obtained very similar associations.

## Discussion

The main results of this study were: 1) Parental SEP measures (parental education and family affluence) were not associated with adolescent drinking; 2) High student weekly income and low academic achievement were associated with higher likelihood of alcohol consumption; 3) The same associations were observed for different drinking measures (weekly binge drinking and weekly alcohol consumption), and for different sexes and age groups; and 4) Although the relationship between SEP indicators and binge drinking varied between countries, in most countries adolescent SEP was associated with alcohol consumption, while parental SEP was not.

### Evaluation of potential limitations

First, alcohol consumption measures were estimated based on self-reported data. As a result, prevalence rates of weekly binge drinking and alcohol consumption may be underestimated due to social desirability bias, as individuals drinking higher amounts of alcohol tend to underestimate their consumption [27]. Besides, it is also possible that some adolescents may exaggerate their alcohol consumption to gain social approval among peers, but this bias may be reduced by the use of a self-completed anonymised questionnaire. However, to attribute our key results to such biases, one would have to assume that they are unrelated to parental SEP but strongly related to own academic achievement and income. This seems unlikely. Moreover, as alcohol use was reported to reflect consumption in the previous year, the frequency of binge drinking and alcohol consumption may also be underestimated due to recall bias.

Second, parental SEP was reported by students instead of parents themselves. As a consequence, we could not ask in detail about the family income or parental occupation status. Moreover, parental education level was the only variable with a relevant proportion of missing data (around 12%). This may introduce bias, as non-response rates tend to be higher among people with low SEP [28]. However, we conducted a sensitivity analysis based on multiple imputation of missing values, and we found that the results were not sensitive to the way in which missing values were dealt with. Finally, the fact that all SEP measures were self-reported may also be a source of social desirability bias, as children may report higher levels of SEP to gain social approval.

### Prevalence of adolescent alcohol use

We found substantial differences in the prevalence of alcohol consumption between countries, with the highest age-adjusted prevalence of weekly binge drinking in Belgium (6.1%) and the lowest in Finland (1.1%). Although the lower prevalence of alcohol use in Finish adolescents may be explained by a steep decline in alcohol use and intoxication in adolescents after 2011 [29], factors related to the cultural context of alcohol use and the different drinking motives in each country [30], as well as alcohol control policies and their enforcement, may explain the relevant differences in prevalence between countries. In this sense, Finish alcohol policies are among the strictest in Europe [31].

The prevalence of alcohol use in all countries is lower than the one estimated in the HBSC survey of 2009–2010 [32]. In Italy for example, we found that the prevalence of weekly alcohol use was 13% (95%CI = 11–14) and 29% (95%CI = 26–33) in girls and boys, respectively, whereas in the HBSC survey the prevalence was 26% (95%CI = 23–29) and 39% (95%CI = 36–42), respectively. A possible explanation for this lower prevalence may be that both studies cover different periods of time and that our data covered single cities while the HBSC survey used representative national samples, which included rural areas where drinking rates tend to be higher than in urban areas [7].

### Parental SEP and adolescent drinking

Our findings suggest that, in general, there is no association between parental SEP and adolescent drinking, which is in line with a review that found no clear pattern in this association [10]. However, other studies have found an association between parental SEP and adolescent drinking in some of these countries [33–35]. When we stratified by country, weekly binge drinking was negatively associated with FAS in Italy, whereas weekly adolescent drinking was positively associated with parental education in Belgium and with FAS in the Netherlands. These results are partly in line with results on the HBSC survey, which found no differences in weekly alcohol use by FAS in Belgium, Finland and Italy, but found a positive association in the Netherlands [20].

Besides, this lack of association between adolescent drinking and parental SEP in the overall sample did not change depending on the alcohol drinking measure. The results from a study in Slovak students were in agreement with ours: no association was found between parental education and both frequency of alcohol use and drunkenness [36].

### Adolescent SEP and adolescent drinking

With respect to the student weekly income, we found a strong positive association: the more available money, the higher the risk of binge drank weekly. This finding is in line with previous studies [22, 37]. In English schoolchildren and US college students, having more money to spend was associated with an increased likelihood of drinking frequently and of binge drinking [22, 37]. This strong relationship implies that alcohol is not only affected by access to social sources, but that the ability to buy from commercial sources is important as well [38]. Adolescents with greater access to money to spend may be able to purchase substances more easily [10, 39]. However, the observed association may also be due to reverse causality as adolescents who drink more often need more money to buy alcohol, they may look for ways to obtain more money (e.g. working or asking for more pocket money) [40]. Probably both processes reinforce each other: the more you drink, the more money you need; and the more money you have, the more you drink. Longitudinal studies that follow young people throughout their adolescence are needed to assess which of these processes contribute most to the alcohol-income association.

High academic achievement appears to be a protective factor for alcohol consumption. Students with higher achievement may be more oriented toward the future and, therefore, be more likely to protect their health. Previous studies found that having high academic goals, such as planning to graduate from university, is a protective factor for substance use among adolescents [41]. Furthermore, adolescents with higher marks are perhaps less likely to engage in after school social activities, as they may spend more time studying and have, consequently, less social involvement. Moreover, negative school experiences (including low academic achievement and low motivation) are known risk factors for substance use [23, 42]. Another possible explanation could be that substance use may also contribute to academic difficulties and thus affect academic performance [18, 43, 44]. Due to the cross-sectional nature of our data, we could not separate social causation explanations (SEP affecting alcohol use) from social selection explanations (alcohol use affecting SEP).

Our results suggest the need for further research using longitudinal data to identify the causal mechanism driving the associations between adolescent drinking and student weekly income and academic achievement, respectively. Besides, research on socioeconomic inequalities in adolescent drinking should include measures of adolescents’ own SEP as they appear to be much more predictive of adolescent alcohol use than parental SEP indicators. A diminished alcohol availability and accessibility in adolescents should be reinforced through stricter alcohol policies, such as increasing alcohol taxes or higher control of alcohol selling, among other effective policies [45–47].

### Conclusions

Low academic achievement and high student weekly income are both strongly associated with higher adolescent drinking. Parental educational level and family affluence are not consistently associated with adolescent alcohol consumption. This suggests that socioeconomic inequalities in adolescent drinking may originate from adolescents’ own situation rather than that of their family. These results imply that health programmes to prevent adolescent drinking in schools should focus especially on children with school problems and low academic performers, just as it has been suggested for tobacco [48]. The important role of student weekly income stresses the importance of greater attention, both in public health research and practice, on how parents could influence their children’s drinking patterns and other health-related behaviour through provision of pocket money.

## Additional files

Additional file 1: **Table S1.** Prevalence ratios (PR) of weekly binge drinking by gender estimated with multilevel Poisson regression models with robust variance among 14–17 years-old students from 6 European cities participating in the SILNE survey, 2013. (DOC 66 kb)

Additional file 2: **Table S2.** Prevalence ratios (PR) of weekly binge drinking by group of age estimated with multilevel Poisson regression models with robust variance among 14–17 years-old students from 6 European cities participating in the SILNE survey, 2013. (DOC 56 kb)

Additional file 3: **Table S3.** Prevalence ratios (PR) of drinking at least one alcoholic beverage per week estimated with multilevel Poisson regression models with robust variance among 14–17 years-old students from 6 European cities participating in the SILNE survey, 2013. (DOCX 14 kb)

Additional file 4: **Table S4.** Prevalence ratios (PR) of drinking at least one alcoholic beverage per week by gender estimated with multilevel Poisson regression models with robust variance among 14–17 years-old students from 6 European cities participating in the SILNE survey, 2013. (DOCX 15 kb)

Additional file 5: **Table S5.** Prevalence ratios (PR) of drinking at least one alcoholic beverage per week by group of age estimated with multilevel Poisson regression models with robust variance among 14–17 years-old students from 6 European cities participating in the SILNE survey, 2013. (DOCX 15 kb)

Additional file 6: **Table S6.** Prevalence of drinking at least one alcoholic beverage per week and prevalence ratios for the socioeconomic position (SEP) variables by country, estimated with multilevel Poisson regression models with robust variance among 14–17 years-old students from 6 European cities participating in the SILNE survey, 2013. (DOCX 16 kb)
