# Supplementary material for: Adolescent alcohol use and parental and adolescent socioeconomic position in six European cities
Source: BMC Public Health. 2017 Aug 8;17:646. doi: 10.1186/s12889-017-4635-7 (PMC5549347; doi:10.1186/s12889-017-4635-7)
Supplement: Supplementary file 1 — Table S1. Prevalence ratios (PR) of weekly binge drinking by gender estimated with multilevel Poisson regression models with robust variance among 14–17 years-old students from 6 European cities participating in the SILNE survey, 2013. (DOC 66 kb) [file 12889_2017_4635_MOESM1_ESM.doc]

**Supplementary Table S1.** Prevalence ratios (PR) of weekly binge drinking by gender estimated with multilevel Poisson regression models with robust variance among 14-17 years-old students from 6 European cities participating in the SILNE survey, 2013.

|  |  | **Female** | | | | |  | **Male** | | | | |
| --- | --- | --- | --- | --- | --- | --- | --- | --- | --- | --- | --- | --- |
|  |  | **Step 2** | |  | **Step 3** | |  | **Step 2** | |  | **Step 3** | |
|  |  | **PR** | **95%CI** |  | **PR** | **95%CI** |  | **PR** | **95%CI** |  | **PR** | **95%CI** |
| **Parental education level** |  |  |  |  |  |  |  |  |  |  |  |  |
| Low level |  | 1 |  |  | 1 |  |  | 1 |  |  | 1 |  |
| Middle level |  | 0.91 | (0.59-1.41) |  | 0.89 | (0.60-1.31) |  | 0.95 | (0.73-1.25) |  | 0.92 | (0.68-1.24) |
| High level |  | 0.88 | (0.55-1.41) |  | 0.87 | (0.58-1.30) |  | 0.92 | (0.56-1.52) |  | 0.88 | (0.55-1.40) |
| **Family Affluence Scale (FAS)** |  |  |  |  |  |  |  |  |  |  |  |  |
| 0 - 2 |  | 1 |  |  | 1 |  |  | 1 |  |  | 1 |  |
| 3 |  | 1.09 | (0.62-1.90) |  | 1.01 | (0.57-1.80) |  | 0.95 | (0.53-1.36) |  | 0.94 | (0.54-1.43) |
| 4 |  | 1.07 | (0.62-1.86) |  | 0.95 | (0.54-1.69) |  | 0.76 | (0.53-1.45) |  | 0.75 | (0.53-1.43) |
| 5 |  | 0.99 | (0.62-1.60) |  | 0.85 | (0.51-1.40) |  | 0.51 | (0.52-1.67) |  | 0.51 | (0.48-1.67) |
| 6 - 7 |  | 1.07 | (0.63-1.82) |  | 0.90 | (0.53-1.54) |  | 0.36 | (0.66-1.75) |  | 0.37 | (0.63-1.65) |
| **Academic achievement** |  |  |  |  |  |  |  |  |  |  |  |  |
| Insufficient (<50%) |  | 1 |  |  | 1 |  |  | 1 |  |  | 1 |  |
| Low (50-59%) |  | 0.37 | (0.16-0.84) |  | 0.38 | (0.15-0.96) |  | 0.85 | (0.51-1.75) |  | 0.88 | (0.51-1.73) |
| Average (60-69%) |  | 0.48 | (0.23-0.98) |  | 0.50 | (0.22-1.15) |  | 0.88 | (0.34-1.68) |  | 0.87 | (0.36-1.59) |
| Good (70-84%) |  | 0.39 | (0.21-0.72) |  | 0.42 | (0.22-0.82) |  | 0.93 | (0.25-1.07) |  | 0.90 | (0.26-0.98) |
| High (>85%) |  | 0.24 | (0.07-0.81) |  | 0.26 | (0.08-0.86) |  | 1.07 | (0.11-1.22) |  | 1.02 | (0.12-1.16) |
| **Student weekly income** |  |  |  |  |  |  |  |  |  |  |  |  |
| 0 - 5 € |  | 1 |  |  | 1 |  |  | 1 |  |  | 1 |  |
| 6 - 10 € |  | 1.57 | (0.82-2.99) |  | 1.58 | (0.82-3.03) |  | 0.68 | (0.42-1.11) |  | 0.69 | (0.43-1.11) |
| 11 - 20 € |  | 2.17 | (1.07-4.43) |  | 2.10 | (1.01-4.37) |  | 1.34 | (0.92-1.96) |  | 1.30 | (0.88-1.92) |
| 21 - 50 € |  | 3.31 | (1.77-6.18) |  | 3.31 | (1.75-6.26) |  | 1.70 | (1.07-2.68) |  | 1.65 | (1.06-2.58) |
| > 50 € |  | 4.14 | (2.22-7.74) |  | 4.19 | (2.19-8.02) |  | 2.60 | (1.84-3.69) |  | 2.63 | (1.84-3.77) |
| **Variability (% change in variability)*** | | |  |  | 0.581 | (17.0) |  |  |  |  | 0.323 | (15.1) |
| Step 2 included weekly binge drinking, one SEP indicator was adjusted by age and migrant background in level 1 and school in level 2. Step 3 included all SEP indicators in one model simultaneously.  *Variability of the empty model (step 1), which included only weekly binge drinking, was 0.699 in female and 0.380 in male. % change in variability was calculated using the following formula: [(variability step 1 - variability current step)/(variability step 1)]x100 | | | | | | | | | | | | |
