# Supplementary material for: Adolescent alcohol use and parental and adolescent socioeconomic position in six European cities
Source: BMC Public Health. 2017 Aug 8;17:646. doi: 10.1186/s12889-017-4635-7 (PMC5549347; doi:10.1186/s12889-017-4635-7)
Supplement: Supplementary file 2 — Table S2. Prevalence ratios (PR) of weekly binge drinking by group of age estimated with multilevel Poisson regression models with robust variance among 14–17 years-old students from 6 European cities participating in the SILNE survey, 2013. (DOC 56 kb) [file 12889_2017_4635_MOESM2_ESM.doc]

**Supplementary Table S2. Prevalence ratios (PR) of weekly binge drinking by group of age estimated with multilevel Poisson regression models with robust variance among 14-17 years-old students from 6 European cities participating in the SILNE survey, 2013.**

|  |  | **14-15-years-old students** | | | | |  | **16-17-years-old students** | | | | |
| --- | --- | --- | --- | --- | --- | --- | --- | --- | --- | --- | --- | --- |
|  |  | **Step 2** | |  | **Step 3** | |  | **Step 2** | |  | **Step 3** | |
|  |  | **PR** | **95%CI** |  | **PR** | **95%CI** |  | **PR** | **95%CI** |  | **PR** | **95%CI** |
| **Parental education level** |  |  |  |  |  |  |  |  |  |  |  |  |
| Low level |  | 1 |  |  | 1 |  |  | 1 |  |  | 1 |  |
| Middle level |  | 0.86 | (0.65-1.14) |  | 0.86 | (0.63-1.18) |  | 0.90 | (0.65-1.25) |  | 0.85 | (0.60-1.19) |
| High level |  | 0.85 | (0.56-1.29) |  | 0.93 | (0.63-1.36) |  | 0.83 | (0.48-1.41) |  | 0.74 | (0.43-1.30) |
| **Family Affluence Scale (FAS)** |  |  |  |  |  |  |  |  |  |  |  |  |
| 0 - 2 |  | 1 |  |  | 1 |  |  | 1 |  |  |  |  |
| 3 |  | 1.21 | (0.60-2.46) |  | 1.19 | (0.58-2.45) |  | 0.76 | (0.47-1.22) |  | 0.78 | (0.48-1.27) |
| 4 |  | 0.90 | (0.51-1.59) |  | 0.88 | (0.51-1.52) |  | 0.92 | (0.57-1.47) |  | 0.89 | (0.54-1.46) |
| 5 |  | 1.14 | (0.64-2.03) |  | 1.13 | (0.63-2.01) |  | 0.76 | (0.46-1.27) |  | 0.73 | (0.42-1.25) |
| 6 - 7 |  | 0.98 | (0.57-1.68) |  | 0.91 | (0.54-1.52) |  | 1.08 | (0.70-1.67) |  | 1.02 | (0.63-1.65) |
| **Academic achievement** |  |  |  |  |  |  |  |  |  |  |  |  |
| Insufficient (<50%) |  | 1 |  |  | 1 |  |  | 1 |  |  |  |  |
| Low (50-59%) |  | 0.67 | (0.32-1.38) |  | 0.66 | (0.32-1.35) |  | 0.83 | (0.40-1.70) |  | 0.79 | (0.37-1.66) |
| Average (60-69%) |  | 0.49 | (0.24-1.02) |  | 0.51 | (0.25-1.06) |  | 0.78 | (0.35-1.74) |  | 0.74 | (0.32-1.70) |
| Good (70-84%) |  | 0.33 | (0.14-0.80) |  | 0.35 | (0.15-0.81) |  | 0.58 | (0.32-1.08) |  | 0.58 | (0.30-1.09) |
| High (>85%) |  | 0.13 | (0.04-0.47) |  | 0.15 | (0.04-0.53) |  | 0.59 | (0.20-1.69) |  | 0.56 | (0.20-1.58) |
| **Student weekly income** |  |  |  |  |  |  |  |  |  |  |  |  |
| 0 - 5 € |  | 1 |  |  | 1 |  |  | 1 |  |  |  |  |
| 6 - 10 € |  | 1.01 | (0.59-1.70) |  | 1.00 | (0.59-1.70) |  | 0.92 | (0.50-1.69) |  | 0.95 | (0.53-1.72) |
| 11 - 20 € |  | 1.91 | (1.13-3.22) |  | 1.82 | (1.09-3.03) |  | 1.42 | (0.94-2.14) |  | 1.42 | (0.94-2.16) |
| 21 - 50 € |  | 2.06 | (1.27-3.34) |  | 1.95 | (1.22-3.12) |  | 2.34 | (1.54-3.57) |  | 2.38 | (1.59-3.57) |
| > 50 € |  | 4.01 | (2.27-7.10) |  | 3.95 | (2.29-6.83) |  | 2.94 | (2.05-4.23) |  | 3.00 | (2.03-4.43) |
| **Variability (% change in variability)*** | | |  |  | 0.562 | (-4.9) |  |  |  |  | 0.248 | (34.3) |
| Step 2 included weekly binge drinking, one SEP indicator was adjusted by gender and migrant background in level 1 and school in level 2. Step 3 included all SEP indicators in one model simultaneously.  *Variability of the empty model (step 1), which included only weekly binge drinking, was 0.535 in younger students and 0.377 in older students. % change in variability was calculated using the following formula: [(variability step 1 - variability current step)/(variability step 1)]x100 | | | | | | | | | | | | |
