# Supplementary material for: Adolescent alcohol use and parental and adolescent socioeconomic position in six European cities
Source: BMC Public Health. 2017 Aug 8;17:646. doi: 10.1186/s12889-017-4635-7 (PMC5549347; doi:10.1186/s12889-017-4635-7)
Supplement: Supplementary file 3 — Table S3. Prevalence ratios (PR) of drinking at least one alcoholic beverage per week estimated with multilevel Poisson regression models with robust variance among 14–17 years-old students from 6 European cities participating in the SILNE survey, 2013. (DOCX 14 kb) [file 12889_2017_4635_MOESM3_ESM.docx]

**Supplementary Table S3.** Prevalence ratios (PR) of drinking at least one alcoholic beverage per week estimated with multilevel Poisson regression models with robust variance among 14-17 years-old students from 6 European cities participating in the SILNE survey, 2013.

|  |  | **Step 2** | |  | **Step 3** | |
| --- | --- | --- | --- | --- | --- | --- |
|  |  | **PR** | **95%CI** |  | **PR** | **95%CI** |
| **Parental education level** |  |  |  |  |  |  |
| Low level |  | 1 |  |  | 1 |  |
| Middle level |  | 1.13 | (0.92-1.39) |  | 1.10 | (0.90-1.34) |
| High level |  | 1.21 | (0.96-1.53) |  | 1.15 | (0.92-1.44) |
| **Family Affluence Scale (FAS)** |  |  |  |  |  |  |
| 0 - 2 |  | 1 |  |  | 1 |  |
| 3 |  | 0.96 | (0.76-1.22) |  | 0.93 | (0.74-1.17) |
| 4 |  | 1.02 | (0.80-1.29) |  | 0.93 | (0.73-1.18) |
| 5 |  | 1.13 | (0.89-1.43) |  | 1.01 | (0.80-1.28) |
| 6 - 7 |  | 1.31 | (1.07-1.61) |  | 1.12 | (0.91-1.36) |
| **Academic achievement** |  |  |  |  |  |  |
| Insufficient (<50%) |  | 1 |  |  | 1 |  |
| Low (50-59%) |  | 0.89 | (0.62-1.28) |  | 0.84 | (0.58-1.22) |
| Average (60-69%) |  | 0.82 | (0.56-1.19) |  | 0.77 | (0.53-1.12) |
| Good (70-84%) |  | 0.64 | (0.45-0.91) |  | 0.61 | (0.44-0.85) |
| High (>85%) |  | 0.44 | (0.28-0.71) |  | 0.43 | (0.27-0.67) |
| **Student weekly income** |  |  |  |  |  |  |
| 0 - 5 € |  | 1 |  |  | 1 |  |
| 6 - 10 € |  | 1.25 | (0.97-1.63) |  | 1.25 | (0.96-1.62) |
| 11 - 20 € |  | 1.84 | (1.47-2.30) |  | 1.77 | (1.40-2.24) |
| 21 - 50 € |  | 2.16 | (1.75-2.65) |  | 2.05 | (1.69-2.50) |
| > 50 € |  | 3.11 | (2.46-3.92) |  | 2.98 | (2.39-3.73) |
| **Variability (% change in variability)*** | | |  |  | 0.539 | (-15.6) |
| Step 2 included weekly alcohol consumption variable (drinking at least one alcoholic beverage per week), one SEP indicator and was adjusted by age, gender and migrant background in level 1 and school in level 2. Step 3 included all SEP indicators in one model simultaneously.  *Variability of the empty model (step 1), which included only weekly alcohol consumption variable (drinking at least one alcoholic beverage per week), was 0.466. % change in variability was calculated using the following formula: [(variability step 1 - variability current step)/(variability step 1)]x100 | | | | | | |
