# Supplementary material for: Adolescent alcohol use and parental and adolescent socioeconomic position in six European cities
Source: BMC Public Health. 2017 Aug 8;17:646. doi: 10.1186/s12889-017-4635-7 (PMC5549347; doi:10.1186/s12889-017-4635-7)
Supplement: Supplementary file 4 — Table S4. Prevalence ratios (PR) of drinking at least one alcoholic beverage per week by gender estimated with multilevel Poisson regression models with robust variance among 14–17 years-old students from 6 European cities participating in the SILNE survey, 2013. (DOCX 15 kb) [file 12889_2017_4635_MOESM4_ESM.docx]

**Supplementary Table S4.** Prevalence ratios (PR) of drinking at least one alcoholic beverage per week by gender estimated with multilevel Poisson regression models with robust variance among 14-17 years-old students from 6 European cities participating in the SILNE survey, 2013.

|  |  | **Female** | | | | |  | **Male** | | | | |
| --- | --- | --- | --- | --- | --- | --- | --- | --- | --- | --- | --- | --- |
|  |  | **Step 2** | |  | **Step 3** | |  | **Step 2** | |  | **Step 3** | |
|  |  | **PR** | **95%CI** |  | **PR** | **95%CI** |  | **PR** | **95%CI** |  | **PR** | **95%CI** |
| **Parental education level** |  |  |  |  |  |  |  |  |  |  |  |  |
| Low level |  | 1 |  |  | 1 |  |  | 1 |  |  | 1 |  |
| Middle level |  | 1.08 | (0.81-1.45) |  | 1.04 | (0.78-1.38) |  | 1.13 | (0.90-1.41) |  | 1.09 | (0.87-1.36) |
| High level |  | 1.16 | (0.89-1.52) |  | 1.10 | (0.84-1.44) |  | 1.18 | (0.87-1.60) |  | 1.12 | (0.85-1.48) |
| **Family Affluence Scale (FAS)** |  |  |  |  |  |  |  |  |  |  |  |  |
| 0 - 2 |  | 1 |  |  | 1 |  |  | 1 |  |  | 1 |  |
| 3 |  | 1.25 | (0.83-1.89) |  | 1.20 | (0.80-1.80) |  | 0.82 | (0.63-1.08) |  | 0.82 | (0.63-1.07) |
| 4 |  | 1.32 | (0.94-1.85) |  | 1.20 | (0.84-1.69) |  | 0.88 | (0.63-1.24) |  | 0.85 | (0.62-1.16) |
| 5 |  | 1.38 | (0.96-1.98) |  | 1.20 | (0.82-1.76) |  | 1.02 | (0.78-1.33) |  | 0.97 | (0.76-1.22) |
| 6 - 7 |  | 1.63 | (1.19-2.23) |  | 1.37 | (1.00-1.87) |  | 1.17 | (0.89-1.54) |  | 1.04 | (0.82-1.33) |
| **Academic achievement** |  |  |  |  |  |  |  |  |  |  |  |  |
| Insufficient (<50%) |  | 1 |  |  | 1 |  |  | 1 |  |  | 1 |  |
| Low (50-59%) |  | 0.59 | (0.34-1.03) |  | 0.58 | (0.32-1.04) |  | 1.03 | (0.69-1.55) |  | 0.97 | (0.65-1.47) |
| Average (60-69%) |  | 0.66 | (0.43-1.00) |  | 0.64 | (0.40-1.02) |  | 0.89 | (0.55-1.45) |  | 0.84 | (0.53-1.35) |
| Good (70-84%) |  | 0.54 | (0.33-0.88) |  | 0.54 | (0.33-0.89) |  | 0.67 | (0.43-1.06) |  | 0.64 | (0.43-0.96) |
| High (>85%) |  | 0.31 | (0.17-0.57) |  | 0.32 | (0.18-0.56) |  | 0.52 | (0.28-0.96) |  | 0.50 | (0.28-0.89) |
| **Student weekly income** |  |  |  |  |  |  |  |  |  |  |  |  |
| 0 - 5 € |  | 1 |  |  | 1 |  |  | 1 |  |  | 1 |  |
| 6 - 10 € |  | 1.56 | (1.00-2.43) |  | 1.53 | (0.99-2.36) |  | 1.12 | (0.90-1.40) |  | 1.12 | (0.90-1.40) |
| 11 - 20 € |  | 2.50 | (1.83-3.43) |  | 2.38 | (1.74-3.26) |  | 1.54 | (1.22-1.95) |  | 1.48 | (1.16-1.91) |
| 21 - 50 € |  | 2.73 | (2.04-3.66) |  | 2.59 | (1.95-3.43) |  | 1.88 | (1.51-2.35) |  | 1.79 | (1.46-2.21) |
| > 50 € |  | 4.34 | (2.99-6.29) |  | 4.11 | (2.84-5.95) |  | 2.54 | (2.01-3.19) |  | 2.46 | (1.96-3.08) |
| **Variability (% change in variability)*** | | |  |  | 0.667 | (-8.9) |  |  |  |  | 0.551 | (-19.8) |
| Step 2 included weekly alcohol consumption variable (drinking at least one alcoholic beverage per week), one SEP indicator and was adjusted by age and migrant background in level 1 and school in level 2. Step 3 included all SEP indicators in one model simultaneously.  *Variability of the empty model (step 1), which included only weekly alcohol consumption variable (drinking at least one alcoholic beverage per week), was 0.613 in female and 0.460 in male. % change in variability was calculated using the following formula: [(variability step 1 - variability current step)/(variability step 1)]x100 | | | | | | | | | | | | |
