# Supplementary material for: Adolescent alcohol use and parental and adolescent socioeconomic position in six European cities
Source: BMC Public Health. 2017 Aug 8;17:646. doi: 10.1186/s12889-017-4635-7 (PMC5549347; doi:10.1186/s12889-017-4635-7)
Supplement: Supplementary file 5 — Table S5. Prevalence ratios (PR) of drinking at least one alcoholic beverage per week by group of age estimated with multilevel Poisson regression models with robust variance among 14–17 years-old students from 6 European cities participating in the SILNE survey, 2013. (DOCX 15 kb) [file 12889_2017_4635_MOESM5_ESM.docx]

**Supplementary Table S5.** Prevalence ratios (PR) of drinking at least one alcoholic beverage per week by group of age estimated with multilevel Poisson regression models with robust variance among 14-17 years-old students from 6 European cities participating in the SILNE survey, 2013.

|  |  | **14-15-years-old students** | | | | |  | **16-17-years-old students** | | | | |
| --- | --- | --- | --- | --- | --- | --- | --- | --- | --- | --- | --- | --- |
|  |  | **Step 2** | |  | **Step 3** | |  | **Step 2** | |  | **Step 3** | |
|  |  | **PR** | **95%CI** |  | **PR** | **95%CI** |  | **PR** | **95%CI** |  | **PR** | **95%CI** |
| **Parental education level** |  |  |  |  |  |  |  |  |  |  |  |  |
| Low level |  | 1 |  |  | 1 |  |  | 1 |  |  | 1 |  |
| Middle level |  | 1.13 | (0.82-1.55) |  | 1.13 | (0.84-1.52) |  | 1.07 | (0.87-1.32) |  | 1.03 | (0.83-1.26) |
| High level |  | 1.22 | (0.88-1.69) |  | 1.23 | (0.91-1.65) |  | 1.08 | (0.81-1.44) |  | 1.01 | (0.75-1.34) |
| **Family Affluence Scale (FAS)** |  |  |  |  |  |  |  |  |  |  |  |  |
| 0 - 2 |  | 1 |  |  | 1 |  |  | 1 |  |  | 1 |  |
| 3 |  | 1.07 | (0.61-1.86) |  | 1.00 | (0.59-1.70) |  | 0.91 | (0.72-1.16) |  | 0.91 | (0.72-1.15) |
| 4 |  | 1.16 | (0.77-1.76) |  | 1.05 | (0.71-1.55) |  | 0.93 | (0.71-1.21) |  | 0.88 | (0.67-1.15) |
| 5 |  | 1.31 | (0.85-2.03) |  | 1.17 | (0.76-1.78) |  | 1.02 | (0.80-1.32) |  | 0.95 | (0.75-1.22) |
| 6 - 7 |  | 1.43 | (0.98-2.08) |  | 1.16 | (0.82-1.65) |  | 1.25 | (1.00-1.57) |  | 1.13 | (0.91-1.40) |
| **Academic achievement** |  |  |  |  |  |  |  |  |  |  |  |  |
| Insufficient (<50%) |  | 1 |  |  | 1 |  |  | 1 |  |  | 1 |  |
| Low (50-59%) |  | 0.89 | (0.52-1.51) |  | 0.86 | (0.51-1.45) |  | 0.92 | (0.64-1.32) |  | 0.88 | (0.60-1.28) |
| Average (60-69%) |  | 0.70 | (0.43-1.13) |  | 0.68 | (0.43-1.10) |  | 0.88 | (0.58-1.35) |  | 0.83 | (0.54-1.30) |
| Good (70-84%) |  | 0.49 | (0.30-0.80) |  | 0.48 | (0.30-0.77) |  | 0.74 | (0.51-1.08) |  | 0.72 | (0.50-1.04) |
| High (>85%) |  | 0.25 | (0.14-0.46) |  | 0.26 | (0.14-0.48) |  | 0.64 | (0.40-1.04) |  | 0.62 | (0.39-0.98) |
| **Student weekly income** |  |  |  |  |  |  |  |  |  |  |  |  |
| 0 - 5 € |  | 1 |  |  | 1 |  |  | 1 |  |  | 1 |  |
| 6 - 10 € |  | 1.49 | (1.02-2.19) |  | 1.45 | (0.98-2.15) |  | 1.15 | (0.86-1.54) |  | 1.16 | (0.86-1.55) |
| 11 - 20 € |  | 2.43 | (1.78-3.30) |  | 2.29 | (1.68-3.12) |  | 1.51 | (1.16-1.97) |  | 1.47 | (1.11-1.95) |
| 21 - 50 € |  | 3.02 | (2.27-4.02) |  | 2.77 | (2.09-3.66) |  | 1.76 | (1.40-2.23) |  | 1.72 | (1.36-2.16) |
| > 50 € |  | 4.98 | (3.41-7.27) |  | 4.65 | (3.22-6.73) |  | 2.38 | (1.90-2.99) |  | 2.31 | (1.82-2.94) |
| **Variability (% change in variability)*** |  |  |  |  | 0.833 | (-36.9) |  |  |  |  | 0.308 | (7.3) |
| Step 2 included weekly alcohol consumption variable (drinking at least one alcoholic beverage per week), one SEP indicator and was adjusted by gender and migrant background in level 1 and school in level 2. Step 3 included all SEP indicators in one model simultaneously.  *Variability of the empty model (step 1), which included only weekly alcohol consumption variable (drinking at least one alcoholic beverage per week), was 0.608 in younger students and 0.333 in older students. % change in variability was calculated using the following formula: [(variability step 1 - variability current step)/(variability step 1)]x100 | | | | | | | | | | | | |
