# Supplementary material for: Adolescent alcohol use and parental and adolescent socioeconomic position in six European cities
Source: BMC Public Health. 2017 Aug 8;17:646. doi: 10.1186/s12889-017-4635-7 (PMC5549347; doi:10.1186/s12889-017-4635-7)
Supplement: Supplementary file 6 — Table S6. Prevalence of drinking at least one alcoholic beverage per week and prevalence ratios for the socioeconomic position (SEP) variables by country, estimated with multilevel Poisson regression models with robust variance among 14–17 years-old students from 6 European cities participating in the SILNE survey, 2013. (DOCX 16 kb) [file 12889_2017_4635_MOESM6_ESM.docx]

**Supplementary Table S6.** Prevalence of drinking at least one alcoholic beverage per week and prevalence ratios for the socioeconomic position (SEP) variables by country, estimated with multilevel Poisson regression models with robust variance among 14-17 years-old students from 6 European cities participating in the SILNE survey, 2013.

|  |  | **Namur (Belgium)** | | |  | **Tampere (Finland)** | | |  | **Hannover (Germany)** | | |  | **Latina (Italy)** | | |  | **Amersfoort (Netherlands)** | | |  | **Coimbra (Portugal)** | | |
| --- | --- | --- | --- | --- | --- | --- | --- | --- | --- | --- | --- | --- | --- | --- | --- | --- | --- | --- | --- | --- | --- | --- | --- | --- |
|  |  | **%** | **PR** | **95%CI** |  | **%** | **PR** | **95%CI** |  | **%** | **PR** | **95%CI** |  | **%** | **PR** | **95%CI** |  | **%** | **PR** | **95%CI** |  | **%** | **PR** | **95%CI** |
| **Parental education level** |  |  |  |  |  |  |  |  |  |  |  |  |  |  |  |  |  |  |  |  |  |  |  |  |
| Low level |  | 11.7 | 1 |  |  | no observations | | |  | 6.3 | 1 |  |  | 17.1 | 1 |  |  | 15.0 | 1 |  |  | 8.5 | 1 |  |
| Middle level |  | 16.5 | 1.23 | (0.95-1.59) |  | 2.8 | 1 |  |  | 7.6 | 1.19 | (0.68-2.09) |  | 20.2 | 1.29 | (0.96-1.73) |  | 10.6 | 0.66 | (0.41-1.05) |  | 7.2 | 0.81 | (0.56-1.17) |
| High level |  | 19.8 | 1.66 | (1.20-2.29) |  | 2.0 | 1.09 | (0.69-1.73) |  | 5.2 | 0.94 | (0.50-1.76) |  | 18.2 | 1.19 | (0.89-1.60) |  | 10.3 | 0.71 | (0.48-1.06) |  | 6.4 | 0.77 | (0.45-1.31) |
| **Family Affluence Scale** |  |  |  |  |  |  |  |  |  |  |  |  |  |  |  |  |  |  |  |  |  |  |  |  |
| 0 - 4 |  | 15.9 | 1 |  |  | 2.5 | 1 |  |  | 6.4 | 1 |  |  | 17.1 | 1 |  |  | 9.7 | 1 |  |  | 6.4 | 1 |  |
| 5 - 7 |  | 20.4 | 1.14 | (0.88-1.46) |  | 2.2 | 0.89 | (0.42-1.89) |  | 6.1 | 1.06 | (0.74-1.53) |  | 20.6 | 1.04 | (0.92-1.18) |  | 11.8 | 1.27 | (1.12-1.44) |  | 7.9 | 1.26 | (0.90-1.77) |
| **Academic achievement** |  |  |  |  |  |  |  |  |  |  |  |  |  |  |  |  |  |  |  |  |  |  |  |  |
| Low (<60%) |  | 21.3 | 1 |  |  | 5.7 | 1 |  |  | 6.9 | 1 |  |  | 40.5 | 1 |  |  | 17.3 | 1 |  |  | 11.1 | 1 |  |
| Average (60-69%) |  | 20.5 | 1.04 | (0.76-1.42) |  | 1.8 | 0.32 | (0.20-0.51) |  | 6.8 | 1.08 | (0.60-1.94) |  | 23.1 | 0.69 | (0.55-0.88) |  | 12.4 | 0.77 | (0.66-0.91) |  | 7.7 | 0.81 | (0.45-1.46) |
| Good (>70%) |  | 13.2 | 0.75 | (0.60-0.93) |  | 0.5 | 0.10 | (0.02-0.42) |  | 5.2 | 1.13 | (0.59-2.16) |  | 13.6 | 0.47 | (0.35-0.63) |  | 7.6 | 0.62 | (0.47-0.82) |  | 4.6 | 0.64 | (0.38-1.07) |
| **Student weekly income** |  |  |  |  |  |  |  |  |  |  |  |  |  |  |  |  |  |  |  |  |  |  |  |  |
| 0 - 5 € |  | 9.9 | 1 |  |  | 0.6 | 1 |  |  | 3.0 | 1 |  |  | 11.1 | 1 |  |  | 4.6 | 1 |  |  | 4.3 | 1 |  |
| 6 - 20 € |  | 16.3 | 1.61 | (0.94-2.75) |  | 1.7 | 2.57 | (0.31-21.49) |  | 3.4 | 1.11 | (0.44-2.86) |  | 20.4 | 1.71 | (1.24-2.37) |  | 5.2 | 0.93 | (0.35-2.47) |  | 7.2 | 1.72 | (1.09-2.71) |
| > 20 € |  | 25.9 | 2.29 | (1.65-3.17) |  | 4.4 | 5.66 | (0.72-44.64) |  | 9.4 | 2.44 | (1.09-5.43) |  | 34.6 | 2.30 | (1.64-3.23) |  | 18.0 | 2.14 | (0.85-5.43) |  | 14.8 | 2.98 | (2.36-3.77) |
| PR: prevalence ratio; 95%CI: 95% confidence interval.  Each model included weekly alcohol consumption variable (drinking at least one alcoholic beverage per week), all SEP indicators and was adjusted by age, gender and migrant background in level 1 and school in level 2. | | | | | | | | | | | | | | | | | | | | | | | | |
